# Reference breast temperature: proposal of an equation

**DOI:** 10.1590/S1679-45082015AO3392

**Published:** 2015

**Authors:** Gladis Aparecida Galindo Reisemberger de Souza, Marcos Leal Brioschi, José Viriato Coelho Vargas, Keli Cristiane Correia Morais, Carlos Dalmaso, Eduardo Borba Neves

**Affiliations:** 1 Universidade Federal do Paraná, Curitiba, PR, Brazil.; 2 Hospital das Clínicas, Faculdade de Medicina, Universidade de São Paulo, São Paulo, SP, Brazil.; 3 Universidade Tecnológica Federal do Paraná, Curitiba, PR, Brazil.

**Keywords:** Breast, Temperature, Metabolism, Thermography, Menstrual cycle

## Abstract

**Objective:**

To develop an equation to estimate the breast reference temperature according to the variation of room and core body temperatures.

**Methods:**

Four asymptomatic women were evaluated for three consecutive menstrual cycles. Using thermography, the temperature of breasts and eyes was measured as indirect reference of core body and room temperatures. To analyze the thermal behavior of the breasts during the cycle, the core body and room temperatures were normalized by means of a mathematical equation.

**Results:**

We performed 180 observations and the core temperature had the highest correlation with the breast temperature, followed by room temperature. The proposed prediction model could explain 45.3% of the breast temperature variation, with variable room temperature variable; it can be accepted as a way to estimate the reference breast temperature at different room temperatures.

**Conclusion:**

The average breast temperature in healthy women had a direct relation with the core and room temperature and can be estimated mathematically. It is suggested that an equation could be used in clinical practice to estimate the normal breast reference temperature in young women, regardless of the day of the cycle, therefore assisting in evaluation of anatomical studies.

## INTRODUCTION

Since the 20^th^ century, publications about infrared thermography for breast imaging have shown controversial results. Compared with some anatomical tests, such as mammography and ultrasound, thermograms were shown to be more relevant for investigation of breast diseases.^(1-3)^ However, in the 21^st^ century, with deeper understanding of neuroendocrine and immunobiological phenomena, the importance of functional studies gave rise to a new approach in breast thermography publications. First of all, the method is not comparable to mammography, since it is a dynamic evaluation of the functional behavior of the skin covering the breast.^(4,5)^

The breast is a gland attached to the dermis and its biological functions, such as microcirculation, perfusion, vascular and inflammatory activity, change constantly and can be documented and quantified by high-resolution thermal imaging.^(6,7)^ Thermal hyperemia, occurring over a few hundredths of a degree, is a new expanded workup approach to traditional clinical inspection that cannot be perceived by the human eye, and produces characteristic images on the skin surface that cannot be accurately quantified as objective temperature data, *e.g*.

Unlike anatomical tests, such as mammography, thermography is a physiological measure that cannot be analyzed in the same context. This is particularly important because, for diagnostic support, not only a qualitative study of thermal distribution is required, but also a quantitative analysis of skin temperature values resulting from the balance between heat transfer from the tissues to the skin surface and from the skin to the environment.^(8)^ However, the skin temperature may vary with the room temperature, as well as with the metabolic activity of tissues and, especially, with the core body temperature.^(9)^

Infrared thermal imaging can only produce reliable and valid results if the technique is compliant with established standards. In medical applications, these standards are based on the physics of heat radiation and thermoregulation of the human body.^(10)^ Thermography can only be used to validate studies of certain diseases if the results published in the literature are comparable, *i.e.*, if they apply the same methodology. But there are no internationally standardized temperature values for diagnostic thermographic evaluation of a given disease, and there is no normal reference range for the breasts, due to major variation in two factors: individual metabolism and room temperature.^(11)^ Each body depending on its size, age, sex, height, weight, body composition, level of physical activity, fasting, hydration and circadian rhythm, has a different core temperature and metabolism, and therefore skin temperature differs by region.^(12-14)^ The literature frequently indicates analysis by thermal gradients, comparing equivalent opposing regions.

Skin temperature may vary 0.5°C for each 1°C increase or decrease in room temperature; the literature recommends that the room temperature be stable with variation of ±1.0°C, preferably ±0.5°C, during the acquisition of thermal images.^(7,14)^ However, although the temperature is kept stable during image acquisition, the room temperature in most published studies varies between 18°C and 26°C.

In clinical practice, it is difficult to establish a testing environment with strict temperature control and consistency every day of the year. The impacting factors are mostly humidity, number and movement of people during the evaluation, room lighting, nearby equipment and heat sources, ventilation, doors opening between examinations, and wall insulation. They all prevent consistent control of room temperature during the examination. Depending on the geographical area and time of year, each location has its own climate changes determining different thermal comfort ranges. When we compare patients from tropical countries and colder places, there is an obvious difference in thermal comfort between different populations. The conditions for thermal comfort depend on the activities developed by individuals, their garments, and environmental variables that allow for heat exchange between body and environment.^(15)^ This also explains the room temperature variation between medical thermography studies of populations from different locations, with adjustment of the temperature based on the patients’ thermal comfort. Colder countries with cooler rooms, and tropical countries with warmer rooms. Individual and environmental variables can influence the thermal comfort of patients. The most important characteristics include metabolism, sex, age, ethnicity, activity level and eating habits.^(16)^

We need to develop an application for thermography in research and clinical practice at different locations that can be validated for investigating diseases by comparison, without the need for rigorous acclimatization to maintain the same consistent temperature during the whole year. That is why it is so important to have the possibility of interpreting these images over a broader room temperature range, respecting the thermal comfort of each patient, and then mathematically correct the temperature to compare with normal parameters and also between the published studies. It is necessary, therefore, to obtain reliable and independent analyzes of the patients’ environmental and metabolic conditions in different data acquisition scenarios.^(17)^

## OBJECTIVES

To develop a prediction equation for the thermal behavior of breasts, regardless of room and core body temperature variations.

## METHODS

Four volunteers with normal breast health were evaluated for 3 consecutive months in search of scientific evidence of temperature changes in the breasts during the monthly menstrual period. The study enrolled four young female volunteers of childbearing age, between 12 and 26 years (16.9±4.7), with no complaints related to the breasts or any other underlying disease, from August 2013 to January 2014. All subjects signed the Informed Consent Form, and the study was conducted according to Resolution 466/2012 of the National Health Council and the recommendations of the Declaration of Helsinki, revised in 2008. The work was approved by the Research Ethics Committee of the *Hospital de Clínicas da Universidade Federal do Paraná*, under number CAAE: 17984413.8.0000.0096 and opinion number 339 545. The healthy volunteers were subjected to daily breast temperature monitoring for 3 months, with a thermographic device (FLIR^®^ T400, USA), in the same room located at the *Núcleo de Pesquisa e Desenvolvimento de Energia Autossustentável da Universidade Federal do Paraná*, at intentionally uncontrolled room temperature.

The device provides images with resolution of 76,800 pixels and sensitivity of 0.05°C, and calculates the skin temperature of the breasts with no need for contact, using the Stephen-Boltzmann equation (Equation 1)


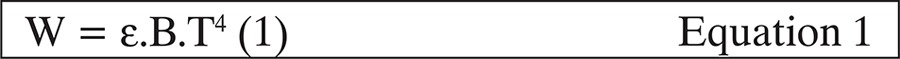


**where**


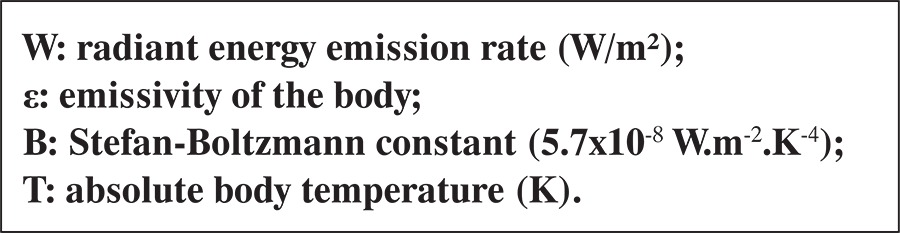


The intensity of the thermal radiation emitted by a body depends on its temperature and ability to emit radiation, which is given by the emissivity of the body. The emissivity is calculated as the relationship between the energy radiated by the body and the energy radiated from a black body at the same temperature, and is 0.98 for the human skin.^(18)^

For measuring the skin temperature of the breasts, the patients previously stood shirtless for 10 minutes in a room without temperature control, according to the local climate (Curitiba (PR), Brazil), always in the morning between 8:00 and 10:00 am, in a closed environment without any airflow and without sweating or shivering.

**Figure 1:**
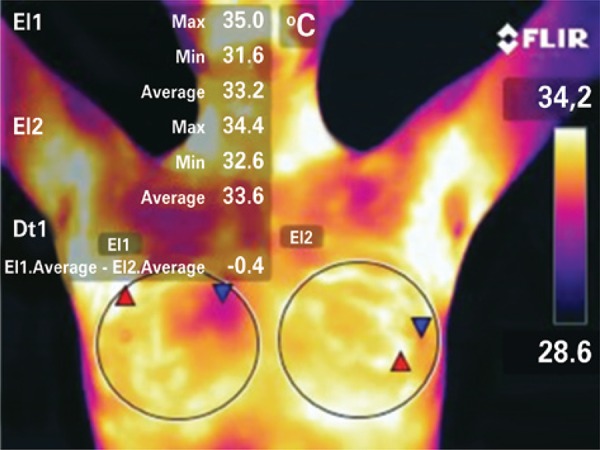
Thermal imaging of the breasts of one of the volunteers on day 9 of the menstrual cycle. Anterior view delimitating the areas of interest to measure the maximum, minimum and average temperatures, and the thermal gradient between them

Anterior views were taken with the arms lifted over the head to expose the entire axillary region, as shown in figure 1. The camera was set vertically, parallel to the body, avoiding any tilting or rotation.^(19)^

**Figure 2:**
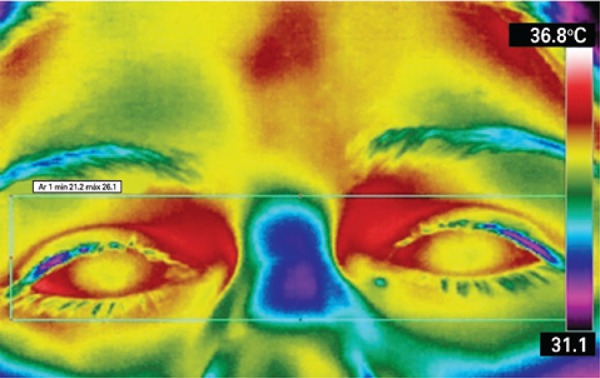
Eye image for measurement of the maximum eye temperature and indirect measurement of the core body temperature by thermography

In this study, the room and core body temperature references defined were, respectively, the minimum temperature recorded on the image background and the maximum eye temperature (MET). The same device was used to record the breast and eye temperatures, which simplifies the methodology using one single measuring instrument and avoiding the standard error between different devices. The core body temperature was indirectly obtained by measuring the MET, more specifically at the medial corner of the eye. This is the warmest area of the face, where supraorbital and suprathroclear arteries emerge to the forehead, as well as direct branches of the ophthalmic artery and the internal carotid, bringing warm blood from the inside (hypothalamus), according to research conducted by Dr. Marc Abreu, a Brazilian working at Yale University. This area is also known as the brain temperature tunnel (BTT).^(19,20)^ The MET was measured as an indirect value closest to the core body temperature (Figure 2).

The room temperature was also indirectly obtained using the value of the thermal image background as reference. The walls of the room where the images were taken was made of cement, and the emissivity used in the formula was corrected to 0.96.

For statistical analysis in this study, we used the paired Student’s “*t*” test, Pearson´s correlation coefficient, standard error of estimate, total error and constant error, as well as linear regression analysis applied to the dataset. The level of statistical significance established was 5% (p<0.05). Statistical analysis was performed with the software Statistical Package for Social Sciences (SPSS) version 21.

## RESULTS

We conducted a total of 180 observations, and 13 images were excluded due to blurring. The mean cycle time was 25.4±3.9 days. The average breast temperature ranged between 32.7°C and 36.4 ^o^C (34.6±0.7°C), with a thermal gradient of 3.7^o^C (Table 1).

**Table 1:** Descriptive data for age, cycle (duration between day 1 of the menstrual period and the day before the next period), and room, core and breast temperatures

	n	Minimum	Maximum	Average	Standard deviation
Age	4	12.0	26.0	16.910	4.788
Cycle time	7	20.0	30.0	25.429	3.909
T					
Right breast	167	32.3	36.5	34.647	0.769
Left breast	167	32.4	36.4	34.696	0.824
Eye (core)	167	34.0	38.0	36.434	0.818
Room	167	15.5	27.6	23.027	2.396
Average breast temperature	167	32.70	36.45	34.6716	0.782

T: temperature in degrees Celsius.

**Figure 3:**
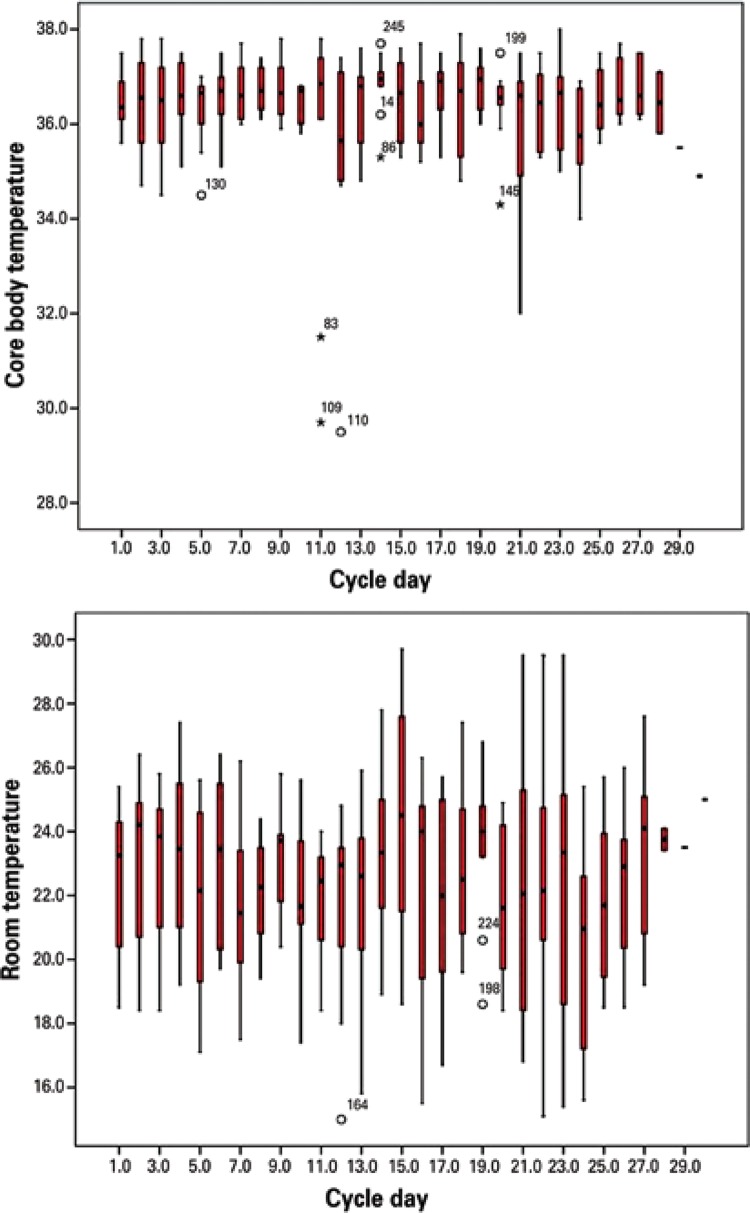
Variability of room and core body temperatures during the menstrual cycle. The core body temperature varied 4°C and the room temperature, 12.1ºC

The room temperature in this study ranged from 15.5°C to 27.6°C (23±2.3°C) and the eye temperature ranged between 34°C and 38°C (36.4±0.8°C), with a respective thermal gradient of 12.1°C and 4°C (Figure 3).

**Figure 4:**
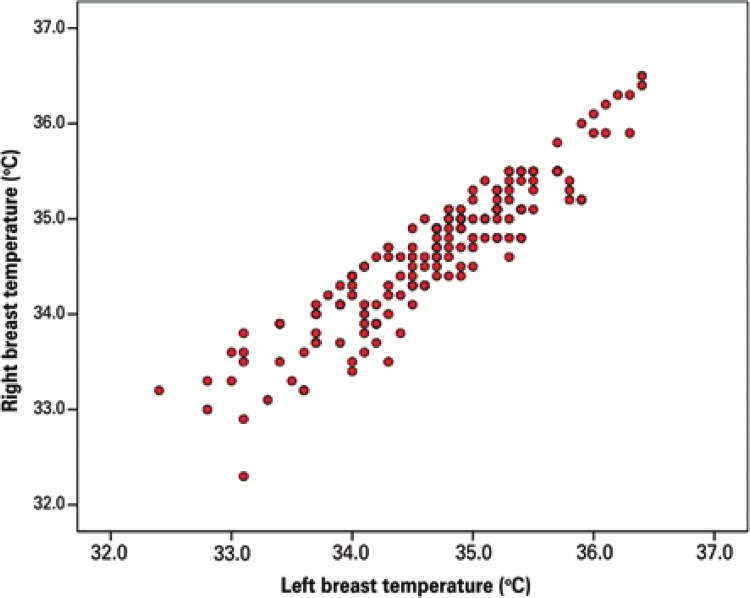
Correlation between the average temperature of the right and left breasts (r=0.927; p<0.001)

There was a high correlation between the average temperature of the right and left breasts (r=0.927, p<0.001) (Figure 4), that is, thermal symmetry was achieved, an expected physiological state in young women with healthy breasts, indicating the reliability of the measuring equipment.

**Figure 5:**
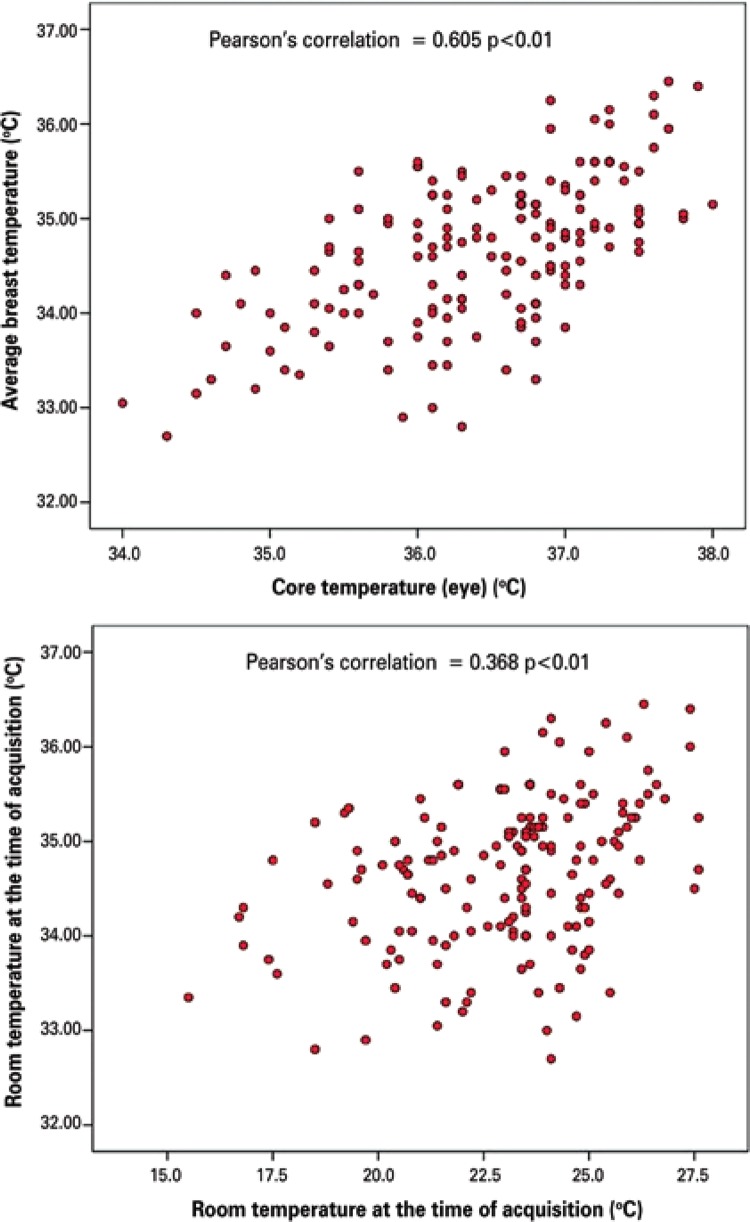
Correlation of average breast temperature with maximum eye temperature (core MET) and room temperature at the time of acquisition

The scatter plot showed there was greater correlation of the average breast temperature with the MET (core), followed by the room temperature (p<0.01) (Figure 5).

The linear regression analysis applied to the dataset in the proposed prediction model had good power to explain the model (r=0.68; R^2^=0.46) (Table 2).

**Table 2:** Results of the linear regression analysis applied to the maximum eye temperature (core MET) and room temperature

Model	Predictors	R	Square R	Adjusted R square	Standard error of estimate
Linear regression	Constant				
	Eye temperature	0.680	0.460	0.453	0.579
	Room temperature				

Based on table 3, we calculated the equation to estimate the breast temperature in relation to the MET (core) and the room temperatures (Equation 2).


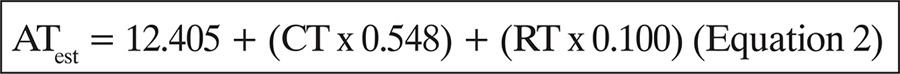


**Table 3:** Coefficients for predictors of breast temperature in relation to the core and room temperatures, obtained from the linear regression analysis applied to breast thermograms

Model for prediction of the average breast T	Not standardized coefficients		Standardized coefficients	t	p value

	B	Standard model	Beta		
Constant	12.405	2.012	-	6.166	0.000
Eye temperature	0.548	0.055	0.573	9.925	0.000
Room temperature	0.100	0.019	0.307	5.326	0.000

T: temperature.

Where AT_est_: estimated average temperature of the breast; CT: maximum eye temperature (core); RT: room temperature.

Equation 2 can also be used to correct the breast temperature to a reference temperature when thermograms are acquired at different temperatures. To do so, simply replace the RT with the desired reference temperature. In this study, the room temperature was indirectly measured based on the background temperature of the thermal image.

## DISCUSSION

The MET had good and significant correlation with the breast temperature (r=0.605). We did not find any studies investigating the correlation between eye or room temperature and the breast temperature. However, correlations of about 0.49 have been published for the maximum and 0.12 for the minimum forehead temperature in relation to the breast temperature.^(20)^ This may suggest that the breasts have a higher correlation with the core body temperature than with the forehead temperature.

In respect to breast and room temperatures, the correlation observed (r=0.368) was regular, but significant. A study investigating the skin temperature variation in unstable environments showed graphic results suggestive of a good correlation between these variables, without specifying the correlation coefficients.^(21)^

Under normal conditions, there is a consistent thermal flow from the inside to the outside of the body. The heat passes on to the environment through the skin. In the trunk, the skin covers a large area of metabolically active muscle and glandular tissue, which continuously produces lots of heat. The temperature in this region is typically determined by the core body temperature through heat transport by conduction.^(22)^Many of the normally inactive veins of the superficial plexus of the breasts becomes active when metabolic activity increases, and the temperature of the avascular part of the skin also increases. This metabolic increase can be indirectly detected based on the core body temperature. To ensure proper functioning of internal organs and the brain, the core temperature and the skin surface temperature should be kept within normal ranges and approximately constant, due to the biochemical reactions necessary for life. The reactions only occur at temperatures close to the optimum value for enzyme activity. Thus, the core body temperature influences the skin temperature by the dynamics of superficial vessels.^(23)^

Although no significant difference was found within a specific phase of the menstrual cycle, the breast temperature had high correlation with the core body temperature at the time of acquisition. In other words, images acquired at the beginning, middle or end of the cycle (month) did not show any specific thermal pattern characterizing higher or lower metabolic activity of the breasts in young women during the menstrual cycle, despite the variation in core temperature. The core body temperature and the room temperature may be masking the effect of hormones on the average breast temperature.

Some studies reported that certain physiological processes in women are related to the different phases of the ovulatory cycle. The most important include changes in gonadotropin, estrogens and progesterone levels in the blood. The diversity of the fluid balances and fluctuations in the core body temperature (rectal), which is approximately 0.4°C, is higher in the luteal phase than in the follicular phase of the cycle.^(24)^ However, this study did not identify any changes in the average breast temperature relative to the menstrual cycle,* i.e.*, the temperatures measured from day 1 of the menstrual period until the day before the next period. Also, there was no increase in the maximum eye temperature during the estimated luteal phase, indicating that the average breast temperature is more closely related to the core MET than to hormonal activity in the breasts. However, this study was not designed to evaluate ovulation in young women, but how the thermal behavior of the breasts varies during the cycles.

The blood palys a role in normalizing the temperature of the organs and muscles, where metabolic heat is generated to be later dissipated to the outside through capillary vessels on the skin. In warm weather, blood converges to the body surface, increasing the temperature. The body starts sweating to cool down the skin and then proceeds to cool down the blood. The opposite happens when the weather is cold. In this case, the blood accumulates heat in the central part of the body, distant from the skin to prevent heat loss.^(25)^ Therefore, the temperature of the skin, including the breasts, is directly affected by the room temperature.

This is why the literature recommends maintaining the temperature of the examination room as constant and uniform as possible,^(26)^ without any air flow exceeding 0.2m/s, which can cause heat loss by forced convection. Nevertheless, a lot of variability was observed in room and core body temperatures measured in a city like Curitiba (PR), with an average daily temperature variation range of 12°C to 13°C. Curitiba (PR) is the coldest capital city in Brazil. The annual average is 16.5 °C (8.4°C to 26.2°C), and it is coldest in July and warmest in February. Therefore, it is not always possible to keep a constant and uniform temperature in a closed environment, pointing to the relevance of finding ways to correct the temperature measured, such as the equation proposed in this study.

The proposed prediction model can explain 45.3% (adjusted R^2^) of the breast temperature variation at variable room temperature, and may be accepted as a way to estimate the reference breast temperature at different room temperatures.

Some tools, such as mammography, ultrasound and MRI, are important resources that allow for morphological evaluation in different breast diseases. Breast thermography is a functional examination proposed as an aid for investigation of new drugs,^(27)^ chemotherapeutic agents,^(28)^ progression monitoring of inflammatory diseases and screening of abnormalities,^(29)^ and could be used in conjunction with clinical and anatomical examinations. Thermography can be used for physiological analysis to detect physiological changes, as well as thermal and vascular abnormalities.^(30)^

One major limitation of this study was failing to use radioimmunoassay techniques to test for hormones or ultrasound images to monitor mature follicular cysts and determine ovulation. Still, this work can serve as a basis for future investigation of the correlation with ovulation, as documented by the methods above.

## CONCLUSION

The average breast temperature in healthy young women was directly related to the core body temperature and the room temperature, and could be mathematically estimated. This study suggests that the use of thermography in medical practice is feasible, by means of an equation to estimate the normal reference breast temperature in young women, regardless of the room temperature and day of the cycle, which could support medical workup and add to anatomical tests. With this method, physicians are able to record the temperature of the breasts and, using a computer program, correct the room and body core temperature variations, normalizing these temperatures without depending on a fixed-temperature environment or prolonged acclimatization. Still, further studies are required to confirm and validate the mathematical relations presented in this article.
