# Supplementary material for: Reference breast temperature: proposal of an equation
Source: Einstein (Sao Paulo). 2015 Oct-Dec;13(4):518–24. doi: 10.1590/S1679-45082015AO3392 (PMC4878624; doi:10.1590/S1679-45082015AO3392)
Supplement: Translation

# Translation: Temperatura de referência das mamas: proposta de uma equação

**Authors:** Gladis Aparecida Galindo Reisemberger de Souza, Marcos Leal Brioschi, José Viriato Coelho Vargas, Keli Cristiane Correia Morais, Carlos Dalmaso, Eduardo Borba Neves

**Affiliations:** 1 Universidade Federal do Paraná, Curitiba, PR, Brasil.; 2 Hospital das Clínicas, Faculdade de Medicina, Universidade de São Paulo, São Paulo, SP, Brasil.; 3 Universidade Tecnológica Federal do Paraná, Curitiba, PR, Brasil.

**Keywords:** Mama, Temperatura ambiente, Metabolismo, Termografia, Ciclo menstrual

## Abstract

**Objetivo:**

Desenvolver uma equação de estimativa da temperatura de referência das mamas, de acordo com a variação das temperaturas ambiente e central.

**Métodos:**

Foram avaliadas quatro mulheres assintomáticas durante três ciclos menstruais consecutivos. Mensuraram-se, por termografia, as temperaturas das mamas e ocular, como referência indireta das temperaturas central e ambiente. Para análise do comportamento térmico durante o ciclo as temperaturas das mamas, foram normalizadas com a temperatura central e ambiente por meio de uma equação matemática.

**Resultados:**

Foram realizadas 180 observações, sendo que a temperatura central apresentou a maior correlação com a temperatura das mamas, seguida da temperatura ambiente. O modelo de predição proposto conseguiu explicar 45,3% da variação da temperatura das mamas com temperatura ambiente variável podendo ser aceito como uma forma de estimar a temperatura de referência das mesmas em diferentes temperaturas ambientais.

**Conclusão:**

A temperatura média das mamas em mulheres saudáveis teve relação direta com a temperatura central e ambiente, e pôde ser estimada matematicamente. Sugere-se que uma equação poderia ser utilizada para estimativa da temperatura de referência da mama normal em mulheres, independente do dia do ciclo e, portanto, auxiliar na avaliação de estudos anatômicos.

## INTRODUÇÃO

Desde o século 20, as publicações a respeito de termografia por infravermelhos para estudo da mama têm suscitado resultados polêmicos. Comparada com exames anatômicos, como a mamografia e a ultrassonografia, prevaleceu a importância destes na pesquisa de enfermidades mamárias.^(1-3)^ Porém no século 21, com entendimento mais aprofundado dos fenômenos neuroendócrinos e imunobiológicos, a importância dos estudos funcionais trouxe uma nova abordagem nas publicações de termografia de mama. Primeiramente, a de que se trata de um método não comparável com a mamografia, pois é o estudo dinâmico do comportamento funcional da pele que recobre a mama.^(4,5)^

A mama é uma glândula anexa da pele, cujas manifestações biológicas, como microcirculação, perfusão, atividade inflamatória e vascular, alteram-se constantemente, e podem ser documentadas e quantificadas por imagem térmica de alta resolução.^(6,7)^ Desse modo, a hiperemia térmica, que ocorre com poucos centésimos de graus, é uma nova abordagem semiológica ampliada da inspeção clínica tradicional, que a visão humana não é capaz de perceber, formando imagens características sobre a superfície cutânea que não podem ser quantificadas de forma precisa, como dados objetivos de temperatura, por exemplo.

Diferentemente de exames anatômicos, como a mamografia, a termografia é uma medida fisiológica que não pode ser analisada no mesmo contexto. Isso é particularmente importante, pois, para apoio diagnóstico, além do estudo qualitativo da distribuição térmica, é utilizada a análise quantitativa dos valores de temperatura da pele resultante do equilíbrio entre a transferência de calor dos tecidos para a superfície cutânea e desta para o ambiente.^(8)^ Porém a temperatura cutânea pode variar com a temperatura ambiente, assim como pela atividade metabólica dos tecidos e, principalmente, da temperatura central.^(9)^

A termografia por imagem infravermelha só pode produzir resultados confiáveis e válidos se a técnica seguir padrões estabelecidos. Em aplicações médicas, esses padrões são baseados na física de radiação de calor e na termorregulação do corpo humano.^(10)^ Para validação dos estudos de determinadas doenças por termografia, é necessário que os resultados publicados na literatura sejam comparáveis entre si, isto é, sigam a mesma metodologia. Porém não existem valores de temperatura padronizados internacionalmente para avaliação termográfica diagnóstica de uma determinada doença e nem de referência normal para as mamas, pois há uma importante variação de dois fatores: o metabolismo individual e a temperatura ambiente.^(11)^ Cada organismo, dependendo de seu tamanho, idade, sexo, altura, peso, composição corporal, atividade física, jejum, hidratação e ciclo circadiano, tem diferente temperatura central e metabolismo corporal e, consequentemente, temperatura cutânea diferente por região.^(12-14)^ Sendo frequentemente indicada, na literatura, a análise por gradientes térmicos, comparando-se regiões opostas equivalentes.

A temperatura cutânea pode variar 0,5°C para cada 1°C que a temperatura ambiente aumenta ou diminui; a literatura recomenda que a temperatura da sala deva ser estável pelo menos com ±1,0°C, preferencialmente ±0,5°C de variação, durante a captura das imagens térmicas.^(7,14)^ No entanto, apesar de manter a temperatura estável durante a captação das imagens, a temperatura da sala na maioria dos estudos publicados é variável entre 18 a 26°C.

Na prática clínica, é difícil estabelecer um ambiente de exames com rígido controle e uniformidade da temperatura todos os dias do ano. Os fatores que mais influenciam são: umidade, número e movimentação de pessoas durante a avaliação, iluminação da sala, equipamentos e fontes de calor próximas, ventilação, abertura de portas entre os exames, e insolação das paredes. Isso dificulta o controle fidedigno da temperatura ambiente durante todo exame. Dependendo da zona geográfica e época do ano, cada localidade possui alterações climáticas próprias, que determinam faixas de conforto térmico diferentes se comparados os pacientes de países tropicais com os de locais mais frios, existindo evidente diferença de conforto térmico entre diferentes populações. As condições do conforto térmico são funções das atividades desenvolvidas pelo indivíduo, de suas vestimentas e das variáveis ambientais que proporcionam trocas de calor entre corpo e ambiente.^(15)^ Tal fator também explica a variação de temperatura da sala entre estudos de termografia médica realizada em povos de localidades distintas, em que a temperatura da mesma procura se adequar à temperatura de conforto térmico do paciente. Países mais frios com salas mais frias e países mais tropicais com salas menos frias. Variáveis individuais e ambientais podem influenciar no conforto térmico do paciente. Destacam-se, entre as características, metabolismo, sexo, idade, raça, atividade e hábitos alimentares.^(16)^

Para desenvolver a aplicação da termografia em pesquisas e na prática clínica em diferentes localidades, bem como sua validação para estudo de doenças por meio de comparação de estudos, sem a dificuldade de se manter o ambiente rigidamente climatizado sempre em uma mesma temperatura fixa uniforme durante o exame no decorrer de todo o ano, seria de grande utilidade a possibilidade de interpretar o exame realizado em uma faixa de temperatura ambiente ampla, respeitando o conforto térmico de cada paciente, e depois poder corrigir matematicamente a temperatura, para poder comparar com parâmetros de normalidade e, assim, entre os estudos publicados. Há a necessidade, então, de se obterem análises confiáveis e independentes das condições ambientais e metabólicas do paciente em diferentes situações de aquisição de dados.^(17)^

## OBJETIVOS

Desenvolver uma equação de estimativa do comportamento térmico das mamas, independente da variação das temperaturas ambiente e central.

## MÉTODOS

Foram avaliadas quatro voluntárias com a saúde das mamas normal, durante 3 meses consecutivos, para identificar evidências cientificas das alteraçãoes de temperaturas nas mamas durante todo o período mensal da mulher. Participaram do estudo quatro voluntárias jovens do sexo feminino, em idade fértil, entre 12 a 26 anos (16,9±4,7), sem queixas em relação às mamas ou qualquer outra doença de base, no período de agosto de 2013 a janeiro de 2014. Todas as participantes do estudo assinaram o Termo de Consentimento Livre e Esclarecido, e a pesquisa foi conduzida de acordo com a resolução 466/2012 do Conselho Nacional de Saúde e as recomendações da Declaração de Helsinque, revisada em 2008. O trabalho foi aprovado pelo Comitê de Ética em Pesquisa sob o número CAAE: 17984413.8.0000.0096 do Hospital de Clínicas da Universidade Federal do Paraná, com número de parecer 339.545. As voluntárias saudáveis foram submetidas a um acompanhamento diário da temperatura das mamas durante 3 meses, com um aparelho termográfico (FLIR^®^ T400, USA) na mesma sala, situada no Núcleo de Pesquisa e Desenvolvimento de Energia Autossustentável da Universidade Federal do Paraná, porém intencionalmente sem controle da temperatura ambiente.

O equipamento permite capturar imagens de 76.800 *pixels *de definição, com sensibilidade de 0,05°C, e calcular a temperatura cutânea das mamas sem contato a partir da equação de Stephen-Boltzman (Equação 1)



**onde**



A intensidade de radiação térmica emitida por um corpo depende da temperatura e da capacidade deste em emitir radiação, que é dada pela emissividade do corpo. O cálculo da emissividade de um corpo é obtido a partir da relação entre a energia irradiada do corpo e a energia irradiada de um corpo negro na mesma temperatura, e corresponde a 0,98 para pele humana.^(18)^

Para aquisição da temperatura cutânea das mamas, a paciente permaneceu previamente em posição ortostática por 10 minutos, com a parte superior despida, em ambiente sem controle de temperatura, conforme clima local (Curitiba, PR, Brasil) sempre pela manhã, entre 8 e 10 horas, em um ambiente fechado, sem corrente de ar e sem que apresentasse sudorese ou tremor.

Foi realizada incidência anterior, com os braços apoiados sobre a cabeça expondo amplamente a região axilar, como demonstrada na figura 1. A câmera ficou verticalmente ajustada, paralela ao corpo, tomando-se cuidado para evitar angulações ou rotações.^(19)^

**Figura 1:** Termografia das mamas de uma das voluntárias, no 9o dia do ciclo menstrual, demonstrando posicionamento de incidência anterior, com delimitação das áreas de interesse para medida das temperaturas máxima, mínima e média, bem como gradiente térmico entre elas

Neste estudo, foram definidas como referência de temperatura ambiente e central respectivamente o registro da temperatura mínima do fundo da imagem e a máxima dos olhos (TMO). Utilizou-se o mesmo equipamento para registro da temperatura das mamas e olhos, facilitando a metodologia por meio de um único instrumento de registro, bem como do erro padrão entre equipamentos para medida de temperatura. Assim, a temperatura central foi obtida indiretamente, mensurando-se a TMO, mais especificamente canto medial dos olhos. Esta é a área mais aquecida da face, da qual emergem para a fronte as artérias supraorbitais e supratrocleares, ramos diretos da artéria oftálmica e carótida interna que trazem sangue aquecido da parte interna (hipotálamo), segundo pesquisa desenvolvida pelo brasileiro Dr. Marc Abreu na Universidade de Yale, denominado também de túnel de temperatura cerebral (BTT).^(19,20)^ Verificou-se então a TMO para obter um valor indireto mais próximo ao da temperatura central do corpo (Figura 2).

**Figura 2:** Imagem ocular para obtenção da temperatura máxima da região ocular e mensuração indireta da temperatura central por termografia

A temperatura ambiente também foi obtida indiretamente utilizando-se o valor térmico do fundo da imagem do termograma como referência. A parede da sala onde foram captadas as imagens era composta de cimento, cuja emissividade utilizada na fórmula foi corrigida para 0,96.

Nesse estudo, utilizaram-se, para análise estatística, o teste *t *de Student pareado, coeficiente de correlação de Pearson, erro padrão de estimativa, erro total e erro constante, além de análise de regressão linear aplicada ao conjunto de dados. O nível de significância estabelecido foi de 5% (p<0,05). A análise estatística dos dados foi executada com o *software Statistical Package for Social Science *(SPSS), versão 21.

## RESULTADOS

Foram realizadas 180 observações ao todo, sendo eliminadas 13 imagens por não apresentarem foco perfeito. A média de dias do ciclo foi de 25,4±3,9. A temperatura média das mamas variou de 32,7 a 36,4°C (34,6±0,7°C), com um gradiente térmico de 3,7°C (Tabela 1).

**Tabela 1:** Dados descritivos da idade, ciclo (duração da etapa entre o primeiro dia da menstruação até o dia que antecede a próxima menorreia), temperatura ambiente, central e das mamas

	n	Mínimo	Máximo	Média	Desvio padrão
Idade	4	12,0	26,0	16,910	4,788
Duração do ciclo	7	20,0	30,0	25,429	3,909
T					
Mama direita	167	32,3	36,5	34,647	0,769
Mama esquerda	167	32,4	36,4	34,696	0,824
Ocular (central)	167	34,0	38,0	36,434	0,818
Ambiente	167	15,5	27,6	23,027	2,396
Média das temperaturas das mamas	167	32,7	36,45	34,6716	0,782

T: temperatura em graus Celsius.

A temperatura ambiente, neste estudo, variou de 15,5 a 27,6°C (23±2,3°C) e a temperatura ocular variou de 34°C a 38°C (36,4±0,8°C), respectivamente 12,1°C e 4°C de gradiente térmico (Figura 3).

**Figura 3:** Variabilidade das temperaturas central e ambiente durante ciclo menstrual. A temperatura central variou 4ºC e a ambiente 12,1ºC

Houve elevada correlação entre a temperatura média das mamas direita com esquerda (r=0,927; p<0,001) (Figura 4), isto é, houve uma simetria térmica, estado fisiológico esperado em mulheres jovens com mamas sadias, indicando fidedignidade na medição realizada pelo equipamento.

**Figura 4:** Correlação entre a temperatura média das mamas direita e esquerda (r=0,927; p<0,001)

O gráfico de dispersão mostrou que houve correlação maior da temperatura média das mamas com a TMO (central), seguida da temperatura ambiente (p<0,01) (Figura 5).

**Figura 5:** Correlação entre temperatura média das mamas com temperatura ocular máxima (TMO central) e temperatura ambiente no momento da aquisição

A análise de regressão linear aplicada ao conjunto de dados no modelo de predição proposto apresentou bom poder de explicação do modelo (r=0,68; R^2^=0,46) (Tabela 2).

**Tabela 2:** Resultados da análise de regressão linear aplicada ao conjunto de dados temperatura máxima ocular (TMO central) e temperatura ambiente

Modelo	Preditores	R	R quadrado	R quadrado ajustado	Erro padrão de estimativa
Regressão linear	Constante				
	Temperatura ocular	0,680	0,460	0,453	0,579
	Temperatura ambiente				

A partir da tabela 3 foi concluída a equação de estimativa da temperatura das mamas em função da TMO (central) e ambiente (Equação 2).

**Tabela 3:** Coeficientes para as variáveis preditoras da temperatura das mamas em função da temperatura central e ambiente, obtidos a partir da análise de regressão linear aplicada aos termogramas de mamas

Modelo para predição da T média das mamas	Coeficientes não padronizados		Coeficientes padronizados	t	Valor de p
	
	B	Modelo padrão	Beta		
Constante	12,405	2,012	-	6,166	0,000
Temperatura ocular	0,548	0,055	0,573	9,925	0,000
Temperatura ambiente	0,100	0,019	0,307	5,326	0,000

T: temperatura.



Onde TM_est_: temperatura média estimada da mama; TC: temperatura máxima ocular (central); TA: temperatura ambiente.

A equação 2 pode também ser utilizada para a correção da temperatura das mamas para uma temperatura de referência quando da aquisição dos termogramas em temperaturas diferentes. Para tanto, basta substituir TA pela temperatura de referência desejada. Neste estudo, foi utilizada uma temperatura ambiente indireta a partir da temperatura do fundo da imagem térmica.

## DISCUSSÃO

A TMO apresentou boa e significativa correlação com a temperatura das mamas (r=0,605). Não foram encontrados estudos que avaliassem a correlação entre a temperatura ocular ou central e a temperatura das mamas. Porém já foram publicadas correlações na ordem de 0,49 para a temperatura máxima da região frontal e 0,12 para a mínima em relação à temperatura das mamas.^(20)^ Assim, pode-se inferir que as mamas apresentam correlação mais elevada com a temperatura central do que com a região frontal.

Em relação à temperatura das mamas com o ambiente, foi observada uma correlação regular (r=0,368), porém significativa. Estudo que investigou a variação da temperatura da pele em ambientes instáveis apresentou resultados gráficos sugerindo boa correlação entre essas variáveis, mas não apresentaram valores exatos dos coeficientes de correlação.^(21)^

Em condições normais, há um fluxo térmico uniforme do interior do corpo para o exterior. A pele é o órgão pelo qual o calor passa para o ambiente. No tronco, a pele cobre uma grande área de massa metabolicamente ativa muscular e glandular, que continuamente produz grande quantidade de calor. A temperatura dessa região é normalmente determinada pela temperatura central por meio do transporte térmico, por condução.^(22)^ Muitas das veias normais inativas do plexo superficial das mamas se tornam ativas com o aumento da atividade metabólica, assim como há aumento da temperatura cutânea da parte avascular. Esse aumento metabólico pode ser avaliado indiretamente pela temperatura central. Para que os órgãos internos e cérebro funcionem corretamente, a temperatura central e a da superfície da pele devem ser mantidas dentro de faixas normais e aproximadamente constantes, em razão das reações bioquímicas necessárias à vida. Estas ocorrem, portanto, próximas a um valor ótimo de temperatura para a atividade das enzimas. Desse modo, a temperatura central influi na temperatura da pele por meio da dinâmica dos vasos superficiais.^(23)^

Apesar de não ter sido constatada uma diferença significativa com um período específico do ciclo menstrual, a temperatura das mamas teve alta correlação com a temperatura central no momento da captura, ou seja, as imagens adquiridas no início, meio ou fim do ciclo (mês) não tiveram um padrão térmico específico suficiente para caracterizar maior ou menor atividade metabólica das mamas em mulheres jovens durante ciclo menstrual, apesar da variação da temperatura central. A temperatura central, bem como a temperatura ambiente, pode ter influenciado, encobrindo o efeito hormonal sobre a temperatura média da mama.

Estudos relatam que certo número de processos fisiológicos no corpo de uma mulher está relacionado com as diferentes fases do ciclo ovulatório. O mais importante destes inclui alterações nos níveis de gonadotropinas, estrogênios e progesterona no sangue. A diversidade dos balanços de água e as flutuações na temperatura central (retal), que é de aproximadamente 0,4°C, são mais elevadas na fase lútea do que na fase folicular do ciclo.^(24)^ No entanto, não foi identificada, neste estudo, variação de temperaturas média das mamas em relação ao período do ciclo menstrual, ou seja, das temperaturas verificadas a partir do primeiro dia da menstruação até o dia que antecedeu a próxima menorreia. Também não foi observada elevação da temperatura máxima ocular na provável fase lútea, o que denota que a temperatura média das mamas tem maior relação com a TMO central do que com a atividade hormonal das mamas. Embora não tenha sido objetivo desta pesquisa avaliar a ovulação de mulheres jovens, e sim a variação do comportamento térmico da mama ao longo dos ciclos.

O sangue exerce a função de normalizador da temperatura dos órgãos e músculos, onde é gerado calor metabólico, que é dissipado nos capilares da pele para o ambiente exterior. Num clima quente, o sangue converge para a superfície do corpo, aumentando a temperatura. O corpo começa a suar para esfriar a pele e inicia-se um processo de esfriamento do sangue. O contrário acontece quando o clima é frio. Neste caso, o sangue acumula calor na parte central do corpo ficando distante da pele e evitando a saída de calor.^(25)^ Portanto, a temperatura da pele, incluindo das mamas, é diretamente afetada pela temperatura ambiente.

Neste sentido, é que a literatura recomenda manter a temperatura da sala de exames o mais constante e uniforme possível,^(26)^ sem correntes de ar e com velocidade não superior a 0,2m/s, que possam provocar perda térmica por convecção forçada. Porém, observou-se grande variabilidade das temperaturas central e ambiente obtidos na prática clínica em uma cidade como Curitiba (PR), que apresenta amplitude térmica média anual que varia entre 12^°^C e 13^°^C. Curitiba (PR), é a capital mais fria do Brasil. A média anual é 16,5°C (8,4°C-26,2°C), mais fria no mês de julho e mais quente em fevereiro. Portanto, nem sempre é possível manter temperatura constante e uniforme, mesmo em um ambiente fechado, o que torna interessantes os meios de correção da temperatura obtida, como, por exemplo, pela equação proposta neste estudo.

O modelo de predição proposto consegue explicar 45,3% (R^2^ ajustado) da variação da temperatura das mamas com temperatura ambiente variável e pode ser aceito como uma forma de estimar a temperatura de referência das mesmas em diferentes temperaturas ambientais.

Ferramentas como mamografia, ultrassonografia e ressonância magnética, são importantes recursos para avaliação das diversas enfermidades da mama, pois permitem avaliação morfológica. A termografia de mamas é um método de análise funcional, que tem sido proposto como auxiliar no estudo de novas medicações,^(27)^ quimioterápicos,^(28)^ acompanhamento evolutivo de doenças inflamatórias, e apoio a triagem de anormalidades,^(29)^ e poderia ser utilizada em conjunto com a avaliação clínica e exames anatômicos. Alterações fisiológicas, anomalias térmicas e vasculares são detectadas por meio da termografia, comprovadamente um método de análise fisiológica.^(30)^

Pode-se citar como uma limitação importante deste estudo a não utilização de técnicas de radioimunoensaio de hormônios ou imagens ultrasonográficas de acompanhamento de cistos foliculares maduros e de corpo lúteo, para a determinação da ovulação. Ainda assim, esses resultados podem servir de base para estudos futuros correlacionando com ovulação documentada pelos métodos anteriores.

## CONCLUSÃO

A temperatura média das mamas em mulheres jovens saudáveis teve relação direta com a temperatura central e do ambiente, e pôde ser estimada matematicamente. Este estudo sugeriu a viabilidade do uso da termografia na prática clínica, por meio de uma equação, para estimativa da temperatura de referência da mama normal em mulheres jovens, independente da temperatura ambiente e dia do ciclo da paciente, auxiliando na avaliação semiológica médica e complementar de estudos anatômicos. Dessa forma, os médicos poderiam registrar as temperaturas das mamas e, por meio de programa de computador, corrigir a variação das temperaturas ambiente e central, normalizando tais temperaturas sem a dependência de um ambiente com temperatura fixa ou um período de aclimatização prolongado. Ainda assim, sugere-se a realização de novos estudos para confirmação e validação das relações matemáticas apresentadas neste trabalho.
